# An Integrative Pause for Healthcare Personnel Challenged by the War: A Personal Perspective

**DOI:** 10.1007/s11920-024-01549-6

**Published:** 2024-10-21

**Authors:** Sameer Kassem, Orit Gressel

**Affiliations:** 1 Rappaport Faculty of Medicine, Technion-Israel Institute of Technology, Haifa, Israel; 2 Internal Medicine Department A, Carmel Medical Center, Haifa, Israel; 3 Integrative Oncology Program, The Oncology Service, Lin, Zebulun, and Carmel Medical Centers, Haifa, Israel

**Keywords:** Integrative Medicine, Internal Medicine, Middle East, War, Healthcare Personnel, Stress, Trauma

## Abstract

**Background:**

As the war in the Middle East intensifies, the support and well-being of healthcare practitioners (HCPs) become paramount to sustaining an effective healthcare system capable of serving the diverse Israeli population.

**Methods:**

In collaboration with the integrative medicine (IM) team, we initiated a treatment intervention with the goal to relieve war-related HCPs concerns.

**Results:**

This trilogy of narratives highlights the multifaceted experiences of HCPs in Israel during times of conflict. The director of internal medicine ward with his conventional medical and leadership expertise, provides stability and order. The IM practitioner offers holistic healing and care. The nurse reflects on her experience of being cared for.

**Conclusions:**

These narratives illustrate the adaptability and resilience of healthcare professionals who navigate their roles with dedication and compassion, ensuring that amidst the strife, the human spirit remains cared for and supported.

## Prologue: Sameer, Internal Medicine Director

Carrying a complex identity at times of war is challenging. On the one hand, identifying with the privileged Israeli society, as an internal medicine specialist with sub-specialty in endocrinology and a PhD in basic science, a director of a highly reputed hospital ward. While on the other hand, a descendant to a Muslim grandfather who uprooted his origin in a Palestinian village in 1948 to become an Israeli citizen, and proud of his grandchildren’s accomplishments on a national and international level. The complexity of our daily lives in Israel and Palestine became common knowledge worldwide. However, not so many are aware of the challenges that face the Israeli society confronted by ethnic, religious, and social elements yielding a mosaic of narratives, beliefs, and values. More than 20% of the Israeli population are Palestinian natives (often regarded as Israeli Arabs by the government) who share deep national, traditional, and economic ties with their relative Palestinians in the West Bank and Gaza. Being part of the Israeli society, working with Israeli Jews on a daily basis amid huge turmoil could sound an impossible mission, but this is our daily reality in the healthcare system, viewed by many as a symbol of mutual respect and tolerance between Jews and Arabs in Israel. Transcending beyond our ethnic and national loyalties to the basic human-to-human loyalty is a matter of deep ethical commitment to many of us, a safe place shared by cross-cultural perspectives and narratives. This, while the Middle Eastern pendulum is continuously swinging between hope and despair. For many of us, the clock stopped ticking on the morning of October 7th, 2023. Despair had dominated the scene.

The day after the Hamas attack in southern Israel, stress and fear were beyond conception. Avoidance was the prevailing tactic that soon became a strategy. Many Palestinian Israelis did not attend their workplaces due to fear and uncertainty. However, as healthcare professionals (HCPs) we did not have the option of deserting our jobs. Our workplaces where Arabs and Jews work and socialize became a bubble of sanity and reason amidst the horrors that were taking place around us. It was, and still is, a call for courage and compassion.

As a head of a department of internal medicine, I am responsible not only for the well-being of my patients and their families but not least for the physical and emotional health of my team. Our staff reflects the Israeli society and is a blend of Jewish and Arab physicians, nurses and other HCPs. At the first days of the war, focusing on “doing” was the safe haven that gathered our staff for a joint purpose: save lives and heal. However, along the next weeks as time passed and the heat of the shock faded slightly, there was a need to move back to “being”, to preserve our “doing” for the days to come.

The concept of introducing integrative medicine (IM) interventions to sooth HCPs emotional and physical concerns and to restore resilience was not alien to us. During the COVID-19 pandemic, we had co-designed with the integrative oncology team in the hospital a model of care, where the hospital personnel, from senior physicians and nurses to the cleaning personnel, were referred to a 30-minute IM treatment during the working hours in the isolated and contaminated departments [1]. This experience of being treated by Chinese needles, touch, and guided imagery, was not easy for a conventional physician as myself, exposed to what seemed at the time as an extraterritorial domain of “spiritual” and “emotional” interventions. There was a lot of uncertainty around many therapeutics that were introduced then at the midst of the pandemic, hence, it was an opportunity to explore, to legitimize our vulnerability and openness to be touched, cared, and healed by our fellow IM practitioners. It was also a time, where across professional disciplines, age, gender, and not less important, with HCPs speaking Hebrew, Arabic, Russian, and Amharic, a shared commitment and motivation to overcome the crisis.

With this experience of collaboration with the IM team, we initiated a call for the hospital administration to renew the IM treatment initiative with the goal to relieve war-related HCPs concerns. This, while acknowledging that some HCPs are willing to share their concerns and others prefer silence, avoidance, and distance. Collaborating with the mental health and social-work teams in the hospital, we opened the gate for HCP referrals that included emotional and sleep concerns as well as those regarded as more ‘physical’ such as pain, fatigue, gain or loss of appetite, and other expressions of the ‘soma’. We offered legitimization of concerns related to the war and the security-tension, while enabling a non-verbal IM intervention or a referral to the mental health team.

## Orit, Integrative Medicine Physician

Sunday morning, October 8th, we sat in our oncology clinic overwhelmed and in shock, as all in Israel. Our IM team works daily with patients undergoing oncology treatments, as we try to support them during times of emotional turmoil and uncertainty. Now the fear and hopelessness were on a national scale. My daughter, son in law and husband were recruited immediately to active duty. My older daughter and her 6-month-old baby moved in with us as they did not have a bomb shelter in their building.

We were thinking with Sameer of re-establishing a resilience program in the hospital ever since working with the hospital staff during the COVID pandemic, so it was just natural for us to want to help now. In the two and a half weeks it took us to get the approval of the ethical committee, we prepared ourselves. Our team attended a 3-day crash course co-instructed by leading trauma psychologists and senior IM physicians on how to approach patients with acute stress disorder (ASD). Also, we reviewed the acupuncture protocol developed by the National Acupuncture Detoxification Association (NADA) [2] and collected donations of equipment, needles, oils and creams from our colleagues abroad. We felt more confident this time around, much more than when working during the pandemic. We couldn’t have been more wrong.

The first HCP I worked with was a resident in the obstetrics and gynecology division complaining of a tic in her eyelid. Acupuncture worked wonders. In the following days, we confronted anxiety, fear, stress, sleep disorder, fatigue, pain and an array of somatic symptoms. Despair was everywhere. It ran through all genders, professions and religions. It felt different than during the pandemic.

## Amal, Emergency Room Nurse

Amal (not her real name), a 36-year-old emergency room (ER) nurse walked into the treatment room all bent over. She was distraught, taking shallow breaths, sobbing. The previous day’s news that her colleague Shai, a nurse called up to military reserve duty, was killed in the war in Gaza had her extremely agitated. He was an ER nurse, married with a young child, and one on the way. A week earlier he was granted a brief leave from the army to accompany his wife to perform amniocentesis. He visited the ward in his military uniform because he had to return to duty as soon as the medical procedure was concluded. For Amal, this unexpected loss was a hard blow. The illusion of being invincible was shattered. She was scared and worried for her brothers, soldiers in the Bedouin battalion of the Israeli army who were also fighting in the war.

The IM practitioner suggested to Amal that she experience an unfamiliar way of “breathing”. She should be gently aware of her breathing, while lengthening exhalation. The IM practitioner suggested that she consider metaphors of water and waves, imagining a journey in tranquil waters. This, while touching her gently, releasing tension from her physical body as well as from her emotional and spiritual spheres. It “worked”. Four minutes following the conclusion of the IM session, Amal left the room, located within the ER area, with a straight posture, calm, composed, ready for the rest of her shift in the ER.

On the day after, Amal shared with us her experience: “*Today I woke up calm*, *with more positive energy. I’m trying to direct my thoughts to think positively*, *too. The treatment was excellent. I came in an emotional flood and a feeling of something heavy pressing on my chest*, *making it difficult for me to breathe. The therapist took me to the sea. I was floating in calm waters. Everything was quiet. The treatment helped me release the tension and the mental stress. With every breath I relaxed my soul. I will continue the breathing exercise she taught me to do*, *because it really helped. Thank you for coming to help us and give us support during this complicated time. I hope we will all know better days*.”

## Closing the Circle

Reflecting on the triad of Sameer, Orit, and Amal, one can imagine the deep meaning of a safe place. Amal represents the wounded healer, her Islamic roots and her medical commitment to patients and colleagues, those who have died and those living. She is the heroine who is brave enough to attend a pilgrimage through suffering toward the unknown. The modern IM healer is the one carrying the metaphoric serpent of touch, whisper, the intention to heal the healer. Asclepius of the year 2024 in Carmel Medical Center, Haifa, is not necessarily Muslim, Jewish, Palestinian, or Israeli. Despite the lack of compassion outside the medical cave, he or she are the hope for conciliation and healing.

## Epilogue

Shai’s wife gave birth to a healthy child at Carmel hospital. Life goes on, but not easy for a fatherless family.

## Data Availability

No datasets were generated or analysed during the current study.
